# Supplementary material for: Theoretical analysis of Polycomb-Trithorax systems predicts that poised chromatin is bistable and not bivalent
Source: Nat Commun. 2019 May 13;10:2133. doi: 10.1038/s41467-019-10130-2 (PMC6513952; doi:10.1038/s41467-019-10130-2)
Supplement: Supplementary file 1 — Supplementary Information [file 41467_2019_10130_MOESM1_ESM.pdf]

Theoretical analysis of Polycomb-Trithorax systems predicts that poised chromatin is bistable and not bivalent

Kim Sneppen and Leonie Ringrose.

Supplementary Material:

Supplementary Figures 1 - 7

Supplementary Table 1

Supplementary Figure 1

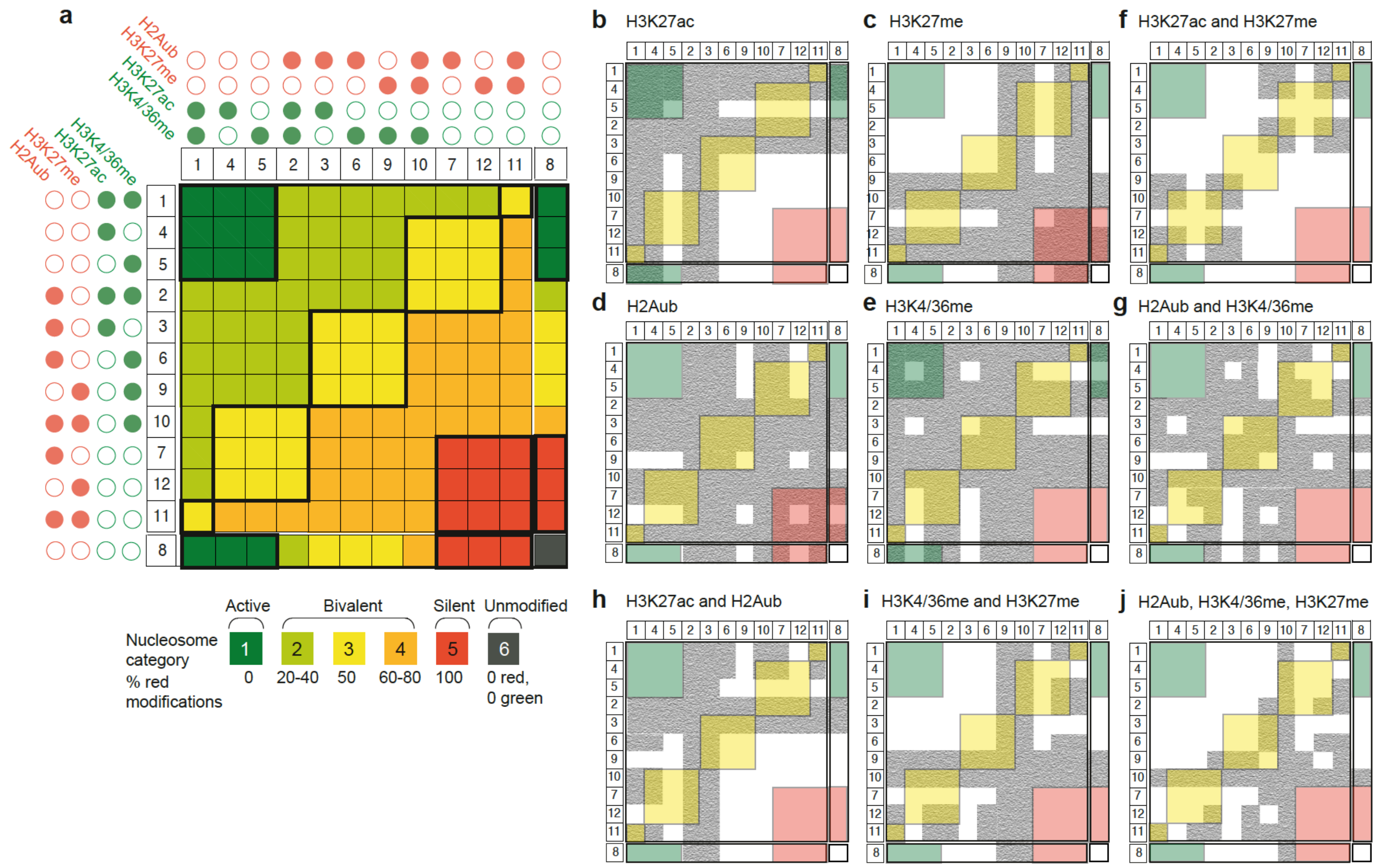

Different nucleosome categories contain specific modifications

(**a**) Half- nucleosomes are shown above and beside the figure with numbers corresponding to Figure 1b, d. Modifications are indicated as closed circles. Red: modifications associated with silencing; green: modifications associated with activation. Whole nucleosomes are colour coded and assigned to categories 1 - 6 according to the proportion of active and silent modifications they contain. (**b-e**) Total possible occurrences of each of the four modifications considered in the model is shown in grey, showing overlap with categories 1 – 6, indicated by transparent coloured boxes. (**f-i**) Total possible occurrences of bivalent nucleosomes carrying double opposing modifications as indicated, showing overlap with categories 1 – 6. (**f**) Bivalent nucleosomes carrying H3K27ac and H3K27me are rare and occur equally frequently in categories 2, 3 and 4. (**g**) Bivalent nucleosomes carrying H2Aub and H3K4/36me are abundant and occur equally frequently in categories 2, 3 and 4. (**h**) Bivalent nucleosomes carrying H3K27ac and H2Aub preferentially occur in category 2. (**i**) bivalent nucleosomes carrying H3K4/K36me and H3K27me preferentially occur in category 4. (**j**) Possible occurrences of nucleosomes carrying H2Aub, H3K4/36me and H3K27me. These preferentially occur in category 4.

Supplementary Figure 2

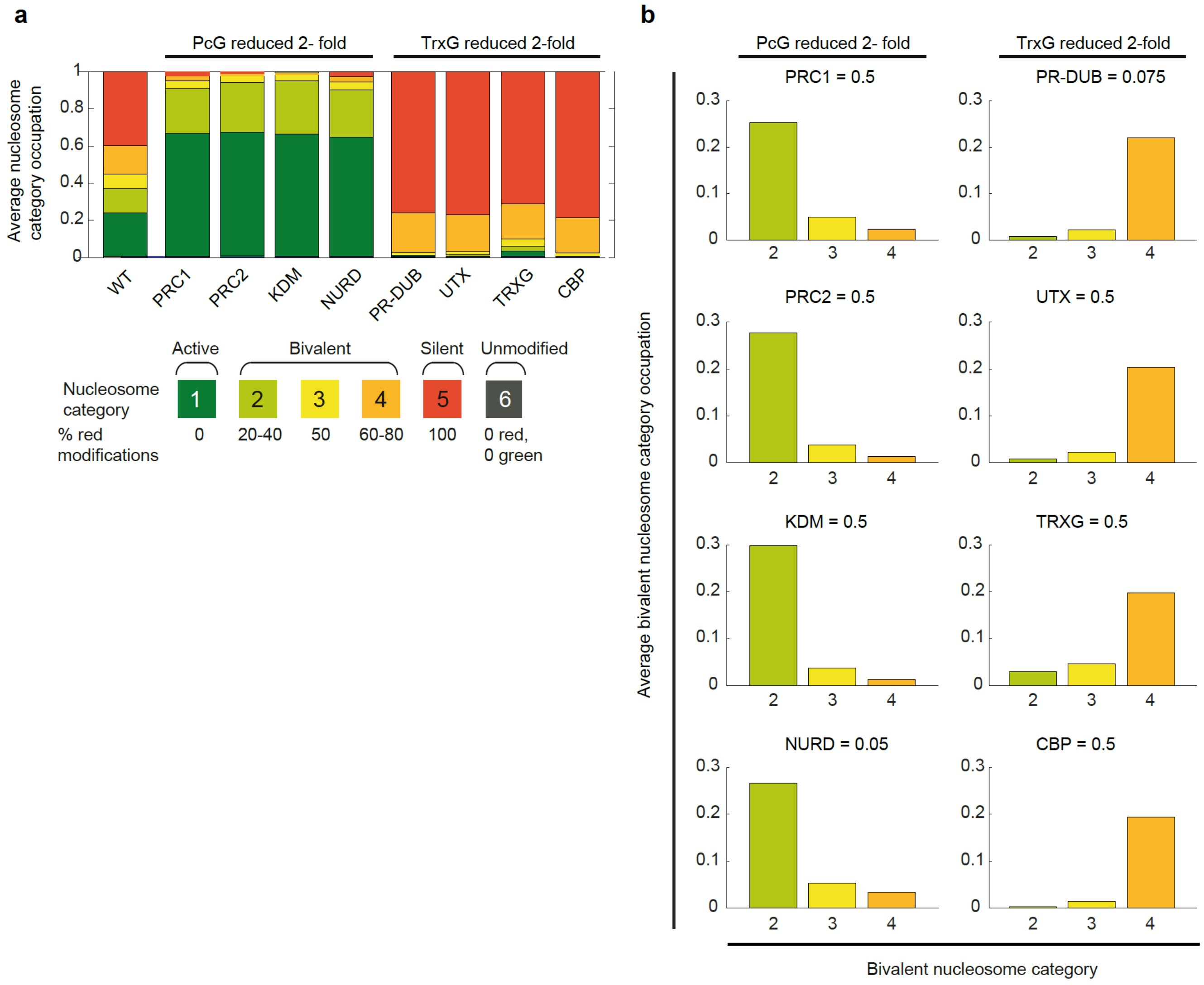

Active and silent system states contain specific bivalent nucleosome categories

(**a**) Nucleosome category occupancy averaged over simulations of 50,000 time units for different conditions. WT: all parameters were set to the values shown in Figure 3b so that the system is bistable. In subsequent columns, single parameters were reduced two – fold, showing that the system flips to an active or silent mode with similar nucleosome category distributions upon different perturbations. (**b**) Occupation of the three bivalent categories (2,3, and 4) as average proportion of total nucleosomes in each category for each of the perturbations shown in (**a**)**,** as indicated. Distinct bivalent categories are consistently associated with active (PcG reduction: category 2 dominates) or silent system states (TrxG reduction: category 4 dominates).

Supplementary Figure 3

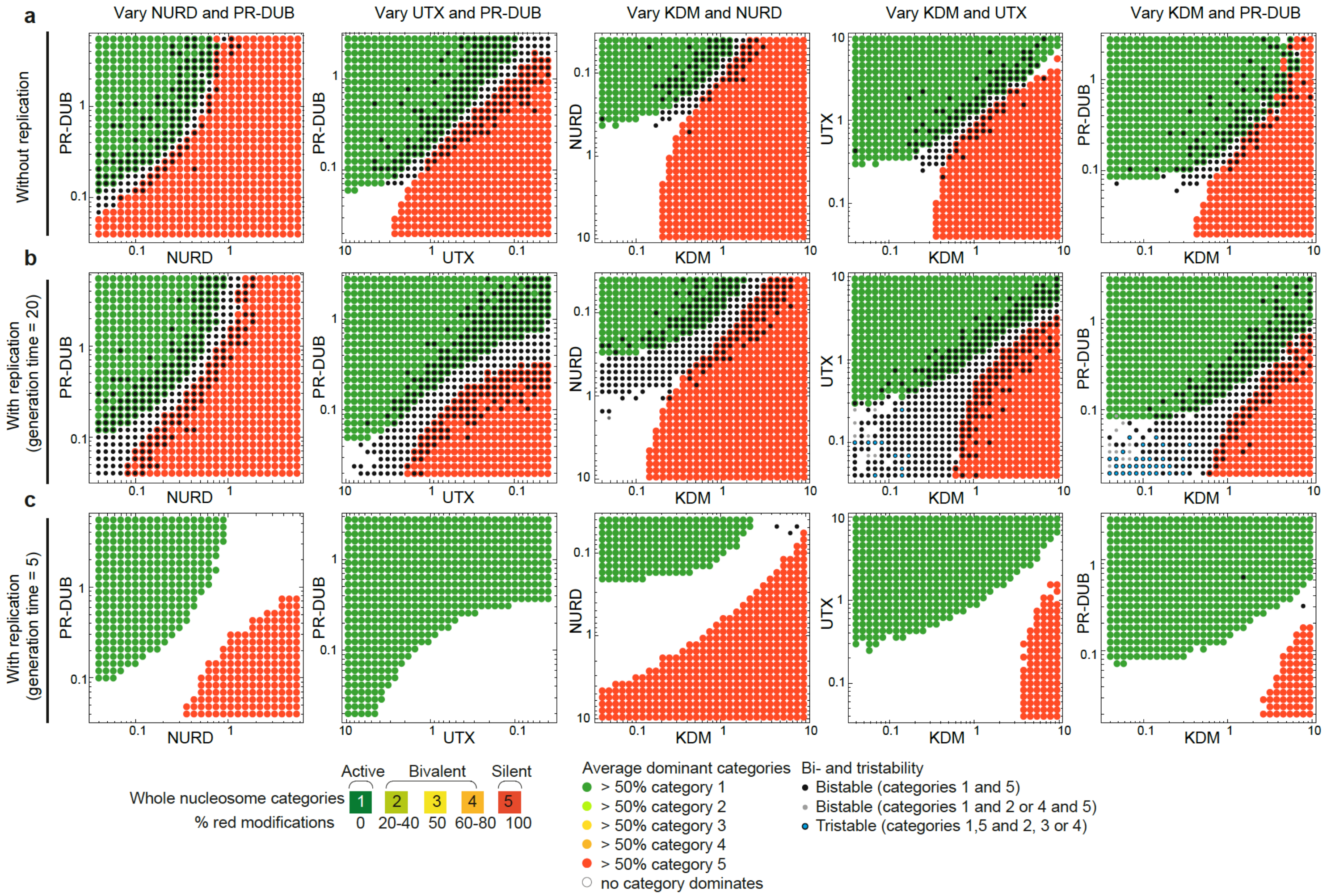

The PcG/TrxG system is robustly bistable

(**a**) System behaviour upon change in pairs of parameters as indicated with all other parameters fixed as in Figure 3b. Each simulation was performed for 50,000 time units and average system state over the entire time course was calculated. Red dots: silent (category 5) modifications dominate (average occupancy of silent modification states larger than 50%). Green dots: active (category 1) modifications dominate. Note that all five modification categories were scored (see legend) but categories 2 - 4 did not dominate under any condition. Black circles: bistability, defined as multiple transitions between situations with more than 60% silent (category 5) nucleosomes and those with more than 60% active (category 1) nucleosomes, each of which has an average lifetime of 40 time units. Grey circles: bistability between the dominant silent or active state and a bivalent state (either category 2 or 4). Blue circles: tristability, with transitions between active, silent and any of the bivalent states (categories 2-4). White areas: none of the above conditions are fulfilled (ie the system switches more rapidly between states and neither state dominates. (**b**) As for (**a**) but with replication once every 20 time units, simulated by resetting each half-nucleosome to the unmodified state 8 with 50% probability. (**c**) As for (**b**) but with replication every 5 time units.

Supplementary Figure 4

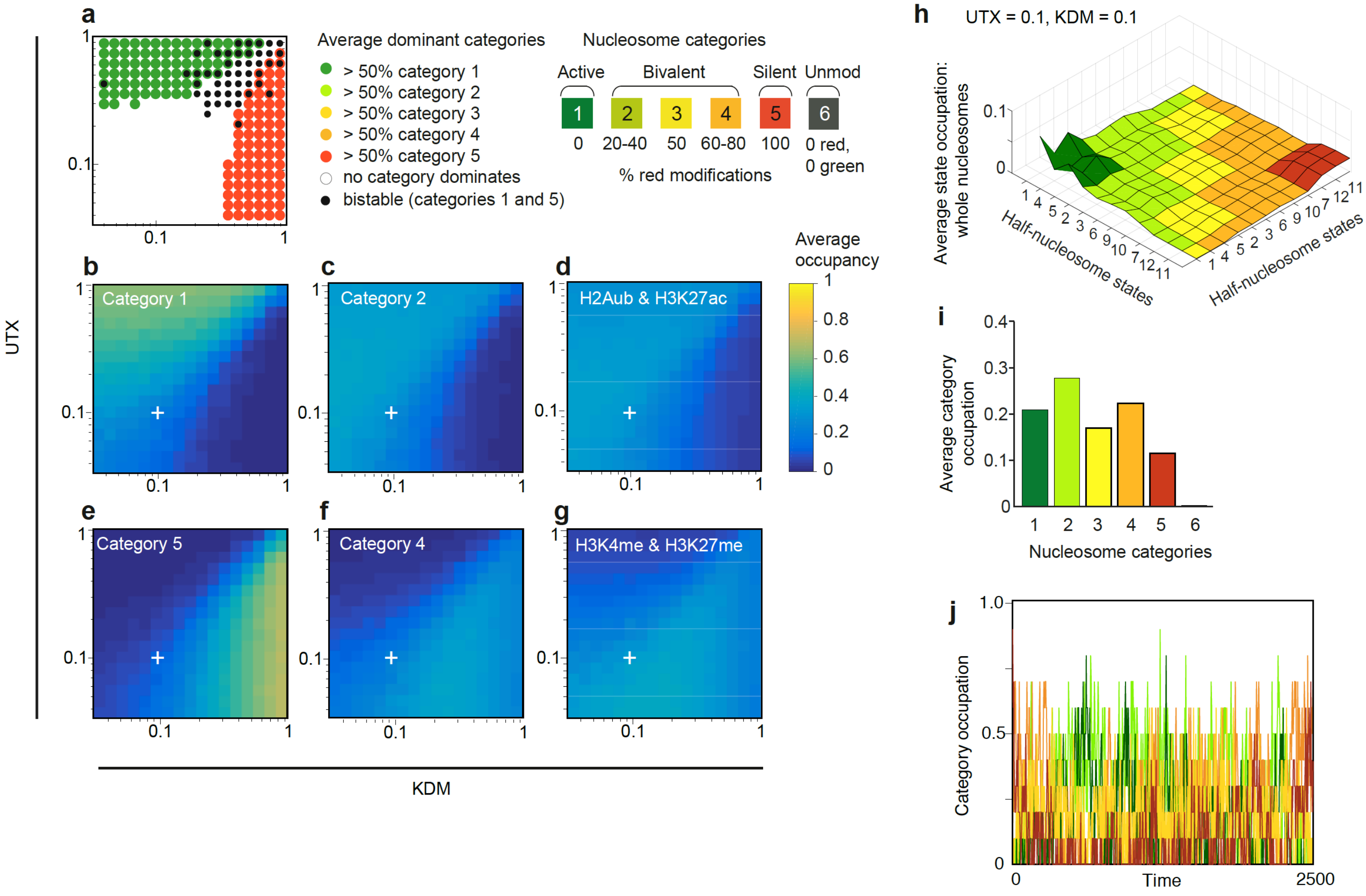

Bivalent nucleosomes do not dominate in the transition zone

(**a**) Zoom of transition zone in Supplementary Figure 3a, panel 4 (KDM and UTX). (**b-g**) For the same parameter values, average occupancies over the simulated time course for different whole nucleosome categories as indicated are shown on the same colour scale (right). (**d**) Probability to find whole nucleosomes carrying both H2Aub and H3K27ac. (**g**) Probability to find whole nucleosomes carrying both H3K4/K36me and H3K27me. (**h-j**) System behaviour when UTX = 0.1 and KDM = 0.1, selected to show the middle of the transition zone, indicated by white crosses on (**b-g**). (**h, i**) Average occupation of each of the 144 nucleosome states (**h**) and categories (**i**) over the time course, showing that the system is still weakly bistable in this zone, although no single category of nucleosomes exceeds 50% on average. (**j**) Simulated time course of dynamics for the same parameter combination, showing that the system switches rapidly between unstable active (mix of categories 1, 2, and 3) and silent (mix of categories 3,4, and 5).

Supplementary Figure 5

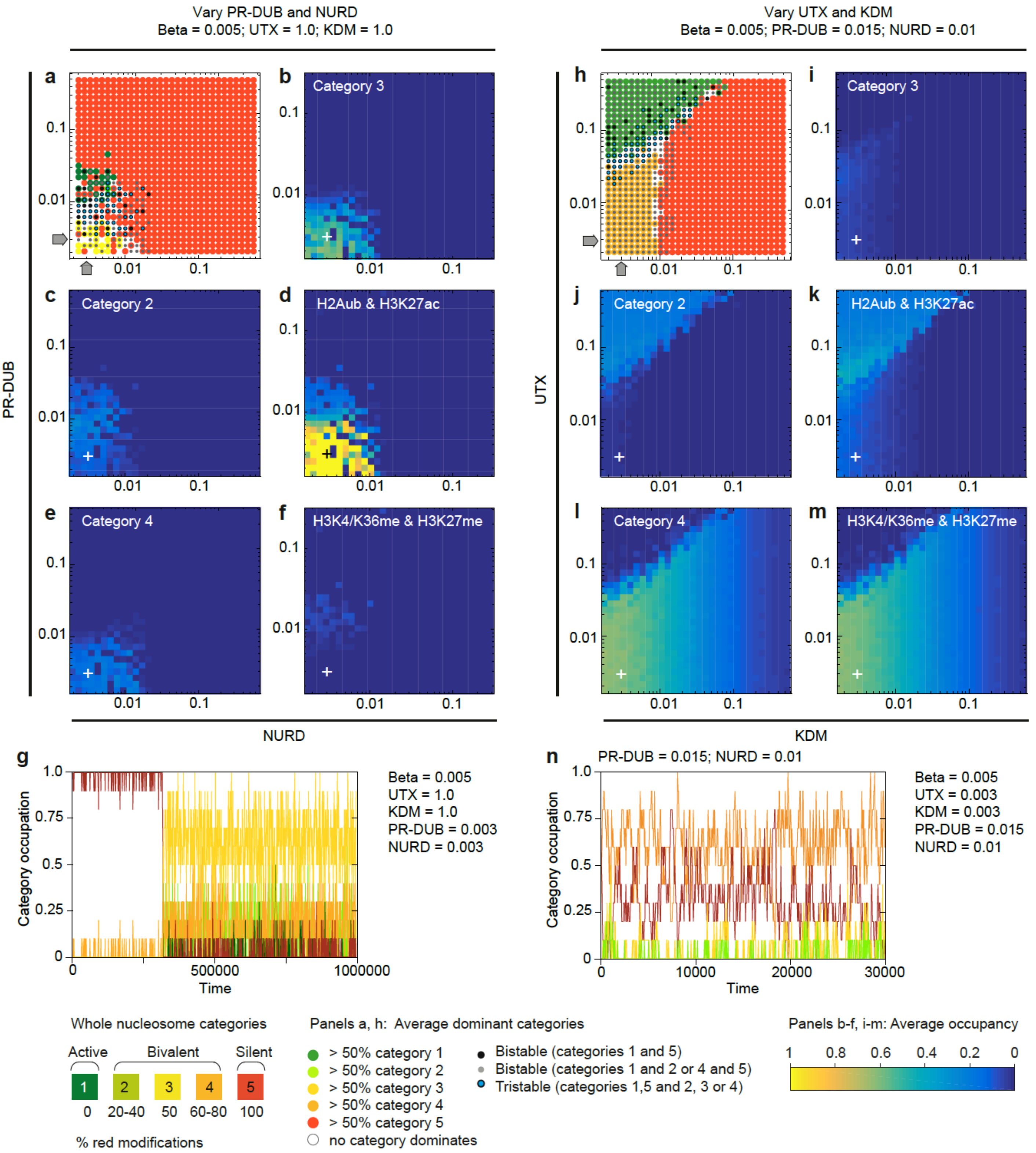

Bivalent chromatin dominates only under extreme conditions

(**a-m**) System behaviour upon change in parameters as indicated with all other parameters fixed as in Figure 3b or as indicated above the figure (See also Supplementary Table 1). Each simulation was performed for 50,000 time units and average system state over the entire time course was

calculated. (**a,h**) Red dots: category 5 dominates (average occupancy of category 5 modification states larger than 50%). Green dots: category 1 dominates. Yellow dots in (**a**): category 3 dominates. Dark yellow dots in (**h**): category 4 dominates. Black circles: bistability, defined as multiple transitions between situations with more than 60% silent (category 5) nucleosomes and those with more than 60% active (category 1) nucleosomes, each of which has an average lifetime of 40 time units. Grey circles, bistability between the dominant silent or active state and a bivalent state. Blue circles: tristability, with transitions between all three states. White areas: none of the above conditions are fulfilled. Grey arrows on (**a,h**) indicate parameter values shown in (**g,n**) and indicated by crosses on (**b-f**) and (**i-m**). (**b-f, i-m**) For the same parameter values, average occupancies over the simulated time course for different whole nucleosome categories as indicated are shown on the same colour scale (bottom right). (**d, k**) Probability to find whole nucleosomes carrying both H2Aub and H3K27ac. (**f, m**) Probability to find whole nucleosomes carrying both H3K4/K36me and H3K27me. (**g, n**) Simulated time course of dynamics of a system of N=20 half nucleosomes with the rate of all transitions in the model set to 1.0, with the exception of Beta, NURD, PR-DUB, UTX and KDM as indicated on the right of each panel. Categories 1 – 5 are indicated according to the colour scale below the Figure. (**g**) Silent states (category 5) initially dominate, and the system then switches permanently to a balanced bivalent (category 3) mode. The most abundant bivalent nucleosomes in this mode carry H2Aub and H3K27ac (**d**). (**n**) Bistable category 4 and 5, dominated by category 4 (silent bivalent). The most abundant bivalent nucleosomes in this mode carry H3K4/K36me and H3K27me (**m**).

Supplementary Figure 6

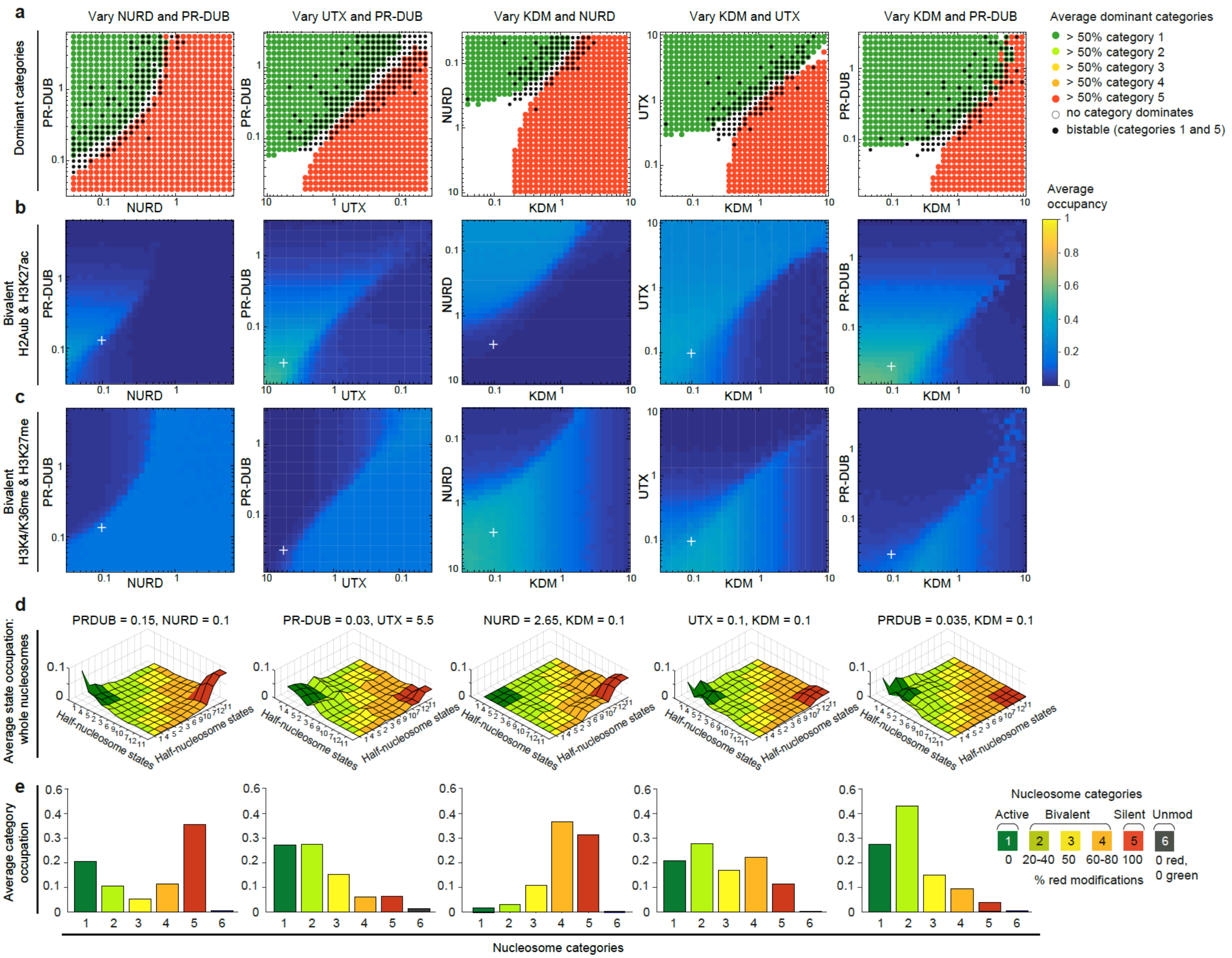

Active and silent chromatin contains distinct bivalent subpopulations

(**a**) Data from Supplementary Figure 3a. System behaviour upon change in pairs of parameters as indicated with all other parameters fixed as in Figure 3b. Each simulation was performed for 50,000 time units and average system state over the entire time course was calculated. Red dots: silent (category 5) modifications dominate (average occupancy of silent modification states larger than 50%). Green dots: active (category 1) modifications dominate. Note that all five modification categories were scored (see legend) but categories 2 - 4 did not dominate under any condition. Black circles: bistability, defined as multiple transitions between situations with more than 60% silent (category 5) nucleosomes and those with more than 60% active (category 1) nucleosomes, each of which has an average lifetime of 40 time units. White areas: none of the above conditions are fulfilled (i.e. the system switches more rapidly between states and no state dominates, see also Supplementary Figure 4j). (**b, c**) For the same parameter values, average occupancies over the simulated time course for specific bivalent whole nucleosomes shown on the same colour scale (below the figure). (**b**) Probability to find whole nucleosomes carrying both H2Aub and H3K27ac.

(**c**) Probability to find whole nucleosomes carrying both H3K4/K36me and H3K27me. Parameter combinations within the transition zone for each plot were chosen as indicated (white crosses on (**b, c**)). (**d, e**) For these parameters, average occupation of each of the 144 nucleosome states (**d**) and categories (**e**) over the time course, showing that the system is still weakly bistable in this transition zone, although no one category of nucleosomes exceeds 50% on average.

Supplementary Figure 7

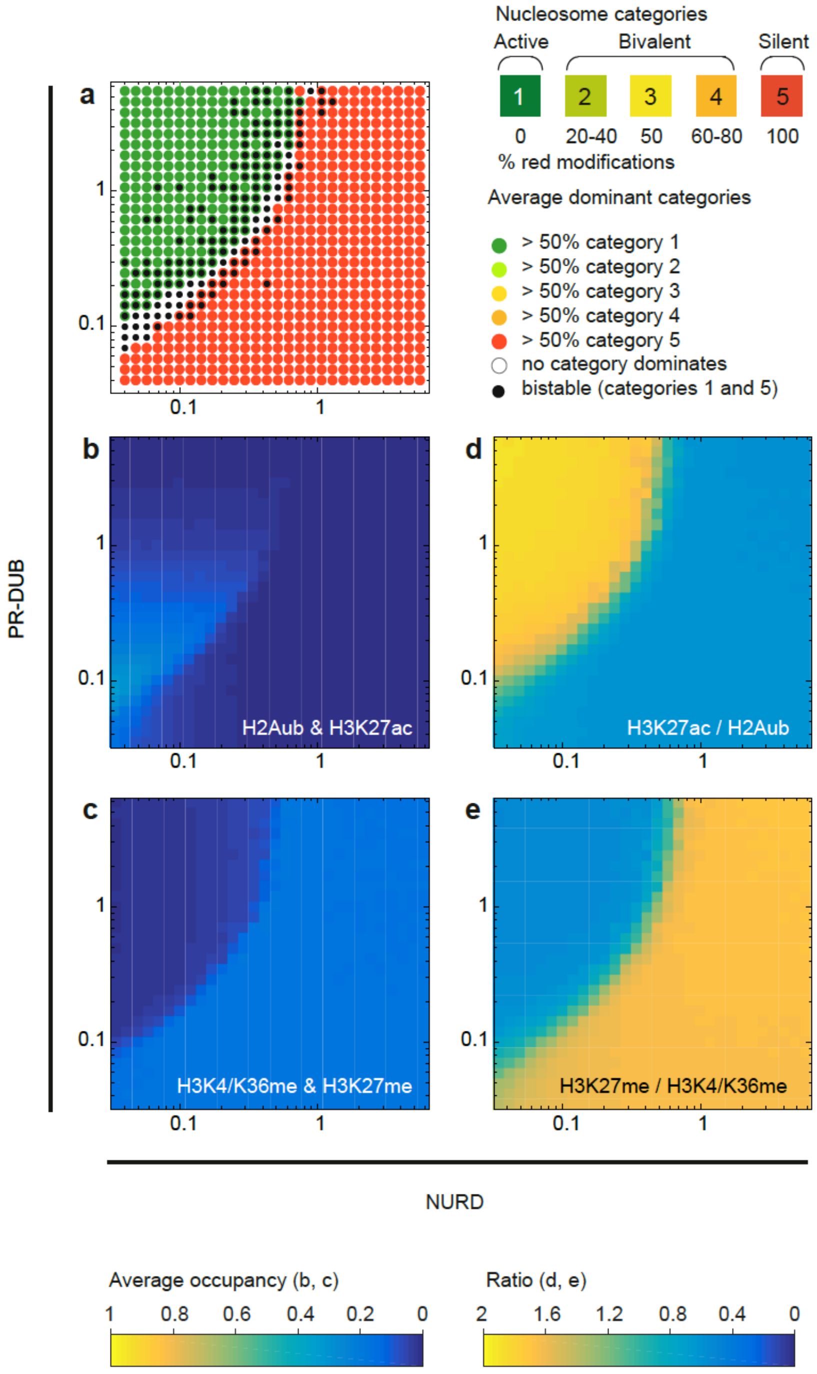

Bivalent chromatin heterogeneity in active and silent system states

(**a**) Data from Figure 3j. (**b, c**) For the same parameter values, average occupancies over the simulated time course for different whole nucleosome categories as indicated are shown on the same colour scale (bottom). (**b**) Probability to find whole nucleosomes carrying both H2Aub and H3K27ac. (**c**) Probability to find whole nucleosomes carrying both H3K4/K36me and H3K27me. (**d, e**) Ratios of pairs of modifications as indicated, calculated using the number of modifications per nucleosome according to Figure 2b.

Supplementary Table 1

Main Figures 3 and 4, Supplementary Figures 2 and 7

	3a,d,g (active)	3b,e,h (bistable)	3c,f,i (silent)	3j,4a, S7	3k	S2	
PRC1	1.0	1.0	1.0	1.0	1.0	1.0	each reduced 2-fold in separate simulations
PRC2	1.0	1.0	1.0	1.0	1.0	1.0	
KDM	1.0	1.0	1.0	1.0	1.0	1.0	
NURD	0.05	0.1	0.2	0.03 - 6.0	0.03 - 6.0	0.1	
PR-DUB	0.15	0.15	0.15	0.03 - 6.0	0.03 - 6.0	0.15	
UTX	1.0	1.0	1.0	1.0	1.0	1.0	
TRXG	1.0	1.0	1.0	1.0	1.0	1.0	
CBP	1.0	1.0	1.0	1.0	1.0	1.0	
Beta	0.1	0.1	0.1	0.1	0.1	0.1	
Replication	no	no	no	no	tgen = 20	no	

Supplementary Figures 2, 3 and 6

S3a, S6a					S3b	S3c
NURD vs PRDUB	UTX vs PRDUB	KDM vs NURD	KDM vs UTX	KDM vs PRDUB		
1.0	1.0	1.0	1.0	1.0		
1.0	1.0	1.0	1.0	1.0		
1.0	1.0	0.06 - 10.0	0.06 - 10.0	0.06 - 10.0		
0.03 - 6.0	0.1	0.03 - 6.0	0.1	0.1		
0.03 - 6.0	0.02 - 3.0	0.15	0.15	0.02 - 3.0	As for S2a	As for S2a
1.0	0.06 - 10.0	1.0	0.06 - 10.0	1.0		
1.0	1.0	1.0	1.0	1.0		
1.0	1.0	1.0	1.0	1.0		
0.1	0.1	0.1	0.1	0.1		
no	no	no	no	no	tgen = 20	tgen = 5

Supplementary Figure 5

	S5a-f	S5h-m	S5g	S5n
PRC1	1.0	1.0	1.0	1.0
PRC2	1.0	1.0	1.0	1.0
KDM	1.0	0.003 - 0.4	1.0	0.003
NURD	0.002 - 0.3	0.01	0.003	0.01
PR-DUB	0.002 - 0.3	0.015	0.003	0.015
UTX	1.0	0.003 - 0.4	1.0	0.003
TRXG	1.0	1.0	1.0	1.0
CBP	1.0	1.0	1.0	1.0
Beta	0.005	0.005	0.005	0.005
Replication	no	no	no	no

Parameters used in Figures
Figure panels are indicated and the list of parameter values, or range of values used in each is indicated. Values correspond to the rates described in Methods section 4. Tgen = 20 or 5: Replication was simulated every 20 or 5 time units.
